# *N*′-[(*E*)-2-Hydroxy­benzyl­idene]-5-methyl­isoxazole-4-carbohydrazide monohydrate

**DOI:** 10.1107/S1600536809048028

**Published:** 2009-11-18

**Authors:** Yan-Xian Jin, Zhen-Zhong Yan, Jian-Guo Yang, Ai-Guo Zhong, Fu-You Pan

**Affiliations:** a School of Pharmaceutical and Chemical Engineering, Taizhou University, Linhai 317000, People’s Republic of China; b Office of Assets Administration, Taizhou University, Linhai 317000, People’s Republic of China

## Abstract

In the structure of the title compound, C_12_H_11_N_3_O_3_·H_2_O, the dihedral angle formed by the benzene and isoxazole rings is 2.03 (8)°. The mol­ecular conformation is stabilized by an intra­molecular O—H⋯N hydrogen bond. In the crystal structure, mol­ecules are linked into a three-dimesional network by inter­molecular N—H⋯O, O—H⋯N and O—H⋯O hydrogen bonds, and by π–π stacking inter­actions involving adjacent benzene and isoxazole rings [centroid–centroid separation = 3.663 (2) Å].

## Related literature

For the biological and coordination properties of hydrazine compounds, see: Molina *et al.* (1994 ▶); Reiter *et al.* (1985 ▶); Sato *et al.* (1998 ▶); Edwards *et al.* (1975 ▶). For the pharmaceutical activity of isoxazole compounds, see: Stevens & Albizati (1984 ▶); El-Gaby *et al.* (2002 ▶). For the synthesis of the title compound, see: Jin *et al.* (2008 ▶). For reference structural data, see: Allen *et al.* (1987 ▶).
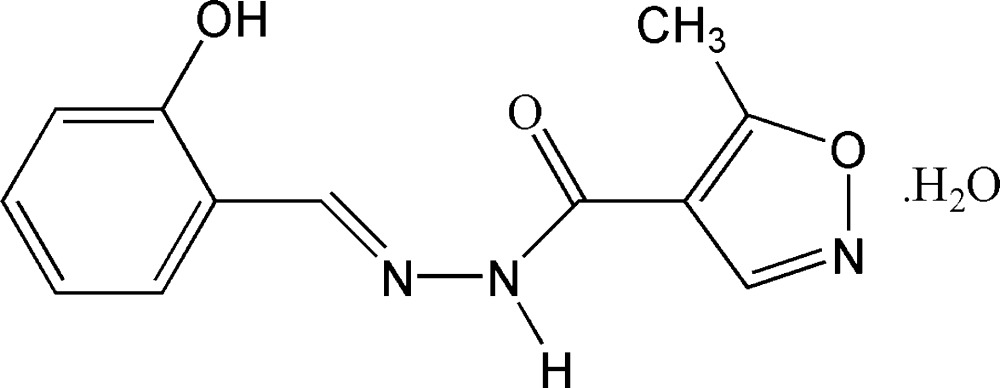


## Experimental

### 

#### Crystal data

C_12_H_11_N_3_O_3_·H_2_O
*M* *_r_* = 263.25
Orthorhombic, 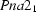

*a* = 12.8783 (6) Å
*b* = 11.3108 (6) Å
*c* = 8.6535 (4) Å
*V* = 1260.50 (11) Å^3^
*Z* = 4
Mo *K*α radiation
μ = 0.11 mm^−1^
*T* = 296 K
0.48 × 0.39 × 0.28 mm

#### Data collection

Bruker APEXII area-detector diffractometer
Absorption correction: multi-scan (*SADABS*; Bruker, 2004 ▶) *T*_min_ = 0.951, *T*_max_ = 0.971
12295 measured reflections
1432 independent reflections
1279 reflections with *I* > 2σ(*I*)
*R*_int_ = 0.039

#### Refinement

*R*[*F* ^2^ > 2σ(*F* ^2^)] = 0.032
*wR*(*F* ^2^) = 0.092
*S* = 0.89
1432 reflections
182 parameters
5 restraints
H atoms treated by a mixture of independent and constrained refinement
Δρ_max_ = 0.14 e Å^−3^
Δρ_min_ = −0.14 e Å^−3^

### 

Data collection: *APEX2* (Bruker, 2004 ▶); cell refinement: *SAINT* (Bruker, 2004 ▶); data reduction: *SAINT*; program(s) used to solve structure: *SHELXS97* (Sheldrick, 2008 ▶); program(s) used to refine structure: *SHELXL97* (Sheldrick, 2008 ▶); molecular graphics: *SHELXTL* (Sheldrick, 2008 ▶); software used to prepare material for publication: *SHELXL97*.

## Supplementary Material

Crystal structure: contains datablocks global, I. DOI: 10.1107/S1600536809048028/rz2385sup1.cif

Structure factors: contains datablocks I. DOI: 10.1107/S1600536809048028/rz2385Isup2.hkl

Additional supplementary materials:  crystallographic information; 3D view; checkCIF report

## Figures and Tables

**Table 1:** Hydrogen-bond geometry (Å, °)

*D*—H⋯*A*	*D*—H	H⋯*A*	*D*⋯*A*	*D*—H⋯*A*
O3—H3*A*⋯N3	0.87 (3)	1.890 (18)	2.617 (2)	145 (2)
N2—H2*B*⋯O1*W*	0.86	2.04	2.8847 (17)	169
O1*W*—H1*W*1⋯N1^i^	0.85 (3)	2.097 (15)	2.9304 (19)	166 (2)
O1*W*—H1*W*2⋯O2^ii^	0.831 (15)	1.955 (15)	2.7850 (17)	176 (2)

Symmetry codes: (i) 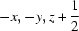; (ii) 
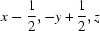
.

**Fig. 1.**
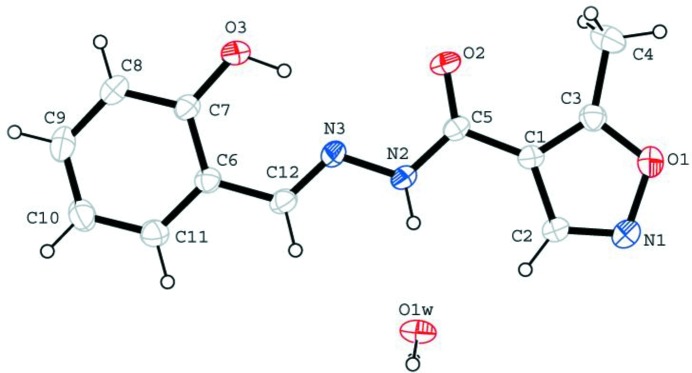
The molecular structure of the title compound. Displacement ellipsoids are drawn at the 30% probability level.

**Fig. 2.**
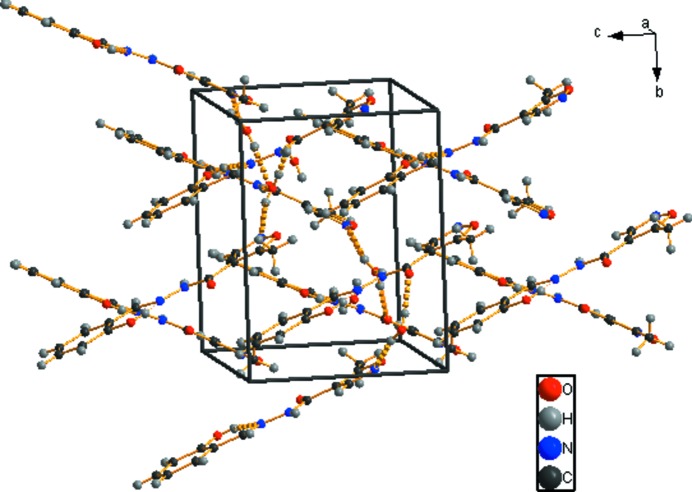
Packing diagram of the title compound. Intermolecular hydrogen bonds are shown as dashed lines.

## supplementary crystallographic information

### Comment

The interest in the study of hydrazine compounds has recently grown due to their biological activities (Molina *et al.*1994; Sato *et al.*1998) and coordination ability (Reiter *et al.*1985; Edwards *et al.*1975). Isoxazole compounds have been widely studied because they exhibit some fungicidal activity, plant-growth regulating activity and antibacterial activity (Stevens *et al.*1984). Some isoxazole derivatives (El-Gaby *et al.*2002) are widely used as insecticides, herbicides and bactericides. However, compounds containing both the hydrazine and isoxazole groups has scarcely been reported. In order to search for more effective antibacterial medicines, we synthesized the title compound and report here its crystal structure.

The molecular structure of the title compounds is shown in Fig. 1. The molecule is almost planar, the dihedral angle between the benzene and the isoxazole rings being 2.03 (8)°. Bond lengths (Allen *et al.*, 1987) and angles in the molecule are within normal ranges. The molecular conformation is enforced by an intramolecular O—H···N hydrogen bond (Table 1). In the crystal packing (Fig. 2), molecules are linked into supramolecular layers by intermolecular O—H···N and N—H···O hydrogen bonds, and by π–π stacking interactions involving adjacent benzene and isoxazole rings, with a centroid-to-centroid separation of 3.663 (2) Å. The layers are further linked by intermolecular O—H···O hydrogen bonds to form a three-dimensional network.

### Experimental

The title compound, C_12_H_13_N_3_O_4_, was synthesized according to the literature method (Jin *et al.*2008). Salicylaldehyde (1.44 ml) was added into a solution of 5-methylisoxazole-4-carbonyl hydrazine (2.0 g, 0.014 mol) in anhydrous ethanol (40 ml). The mixture was refluxed for 2 h, then the precipitate was collected by filtration and washed with water, chloroform and ethanol. The product was recrystallized from ethanol, then dried under reduced pressure (yield 84.5%). Pink block-shaped crystals were obtained by slow evaporation of a dimethylformamide solution.

### Refinement

The water and hydroxyl H atoms were located in a difference Fourier map and isotropically refined with the O—H distance restrained to 0.86 (1) Å. All other H atoms were positioned geometrically and allowed to ride on their parent atoms, with C—H = 0.93–0.96 Å, N—H = 0.86 Å, and with *U*_iso_(H) = 1.2 U_eq_(C, N) or 1.5 U_eq_(C) for methyl H atoms. In the absence of significant anomalous scattering effects, Friedel pairs were merged in the final refinement.

### Crystal data

**Table:**

C_12_H_11_N_3_O_3_·H_2_O	*F*(000) = 552
*M**_r_* = 263.25	*D*_x_ = 1.387 Mg m^−^^3^
Orthorhombic, *P**n**a*2_1_	Mo *K*α radiation, λ = 0.71073 Å
Hall symbol: P 2c -2n	Cell parameters from 6440 reflections
*a* = 12.8783 (6) Å	θ = 2.4–27.0°
*b* = 11.3108 (6) Å	µ = 0.11 mm^−^^1^
*c* = 8.6535 (4) Å	*T* = 296 K
*V* = 1260.50 (11) Å^3^	Block, pink
*Z* = 4	0.48 × 0.39 × 0.28 mm

### Data collection

**Table:**

Bruker APEXII area-detector diffractometer	1432 independent reflections
Radiation source: fine-focus sealed tube	1279 reflections with *I* > 2σ(*I*)
graphite	*R*_int_ = 0.039
ω scans	θ_max_ = 27.0°, θ_min_ = 2.4°
Absorption correction: multi-scan (*SADABS*; Bruker, 2004)	*h* = −16→16
*T*_min_ = 0.951, *T*_max_ = 0.971	*k* = −12→13
12295 measured reflections	*l* = −10→11

### Refinement

**Table:**

Refinement on *F*^2^	Primary atom site location: structure-invariant direct methods
Least-squares matrix: full	Secondary atom site location: difference Fourier map
*R*[*F*^2^ > 2σ(*F*^2^)] = 0.032	Hydrogen site location: inferred from neighbouring sites
*wR*(*F*^2^) = 0.092	H atoms treated by a mixture of independent and constrained refinement
*S* = 0.89	*w* = 1/[σ^2^(*F*_o_^2^) + (0.0725*P*)^2^ + 0.135*P*] where *P* = (*F*_o_^2^ + 2*F*_c_^2^)/3
1432 reflections	(Δ/σ)_max_ = 0.097
182 parameters	Δρ_max_ = 0.14 e Å^−^^3^
5 restraints	Δρ_min_ = −0.14 e Å^−^^3^

### Special details

**Table:**

Geometry. All e.s.d.'s (except the e.s.d. in the dihedral angle between two l.s. planes) are estimated using the full covariance matrix. The cell e.s.d.'s are taken into account individually in the estimation of e.s.d.'s in distances, angles and torsion angles; correlations between e.s.d.'s in cell parameters are only used when they are defined by crystal symmetry. An approximate (isotropic) treatment of cell e.s.d.'s is used for estimating e.s.d.'s involving l.s. planes.
Refinement. Refinement of *F*^2^ against ALL reflections. The weighted *R*-factor *wR* and goodness of fit *S* are based on *F*^2^, conventional *R*-factors *R* are based on *F*, with *F* set to zero for negative *F*^2^. The threshold expression of *F*^2^ > σ(*F*^2^) is used only for calculating *R*-factors(gt) *etc*. and is not relevant to the choice of reflections for refinement. *R*-factors based on *F*^2^ are statistically about twice as large as those based on *F*, and *R*- factors based on ALL data will be even larger.

### Fractional atomic coordinates and isotropic or equivalent isotropic displacement parameters (Å^2^)

**Table:**

	*x*	*y*	*z*	*U*_iso_*/*U*_eq_	
N1	0.14390 (16)	−0.00283 (18)	0.3335 (3)	0.0565 (5)	
N2	0.24031 (12)	0.15708 (15)	0.7518 (2)	0.0421 (4)	
H2B	0.1744	0.1523	0.7368	0.051*	
N3	0.27882 (14)	0.20055 (16)	0.8883 (2)	0.0416 (4)	
O1W	0.01706 (11)	0.16789 (14)	0.7294 (2)	0.0559 (4)	
H1W1	−0.026 (2)	0.123 (2)	0.776 (4)	0.084*	
H1W2	−0.019 (2)	0.2255 (19)	0.704 (4)	0.084*	
O1	0.24526 (12)	−0.01962 (14)	0.2774 (2)	0.0550 (4)	
O2	0.40155 (10)	0.13200 (14)	0.6549 (2)	0.0517 (4)	
O3	0.43156 (13)	0.25524 (19)	1.0747 (2)	0.0648 (5)	
C1	0.26059 (14)	0.06858 (16)	0.5035 (3)	0.0373 (4)	
C2	0.15530 (16)	0.0488 (2)	0.4659 (3)	0.0467 (5)	
H2A	0.1002	0.0704	0.5294	0.056*	
C3	0.31271 (16)	0.02328 (18)	0.3808 (3)	0.0442 (5)	
C4	0.42389 (18)	0.0114 (3)	0.3423 (4)	0.0716 (8)	
H4A	0.4309	−0.0251	0.2427	0.107*	
H4B	0.4575	−0.0367	0.4189	0.107*	
H4C	0.4555	0.0882	0.3404	0.107*	
C5	0.30730 (14)	0.12213 (16)	0.6423 (3)	0.0376 (4)	
C6	0.25002 (15)	0.27711 (17)	1.1395 (3)	0.0390 (4)	
C7	0.35615 (16)	0.29084 (19)	1.1731 (3)	0.0443 (5)	
C8	0.3861 (2)	0.3415 (2)	1.3120 (3)	0.0578 (6)	
H8A	0.4563	0.3500	1.3347	0.069*	
C9	0.3126 (2)	0.3794 (2)	1.4164 (3)	0.0583 (6)	
H9A	0.3336	0.4143	1.5086	0.070*	
C10	0.2090 (2)	0.3664 (2)	1.3859 (3)	0.0568 (6)	
H10A	0.1598	0.3912	1.4576	0.068*	
C11	0.17799 (17)	0.31606 (19)	1.2483 (3)	0.0488 (5)	
H11A	0.1075	0.3080	1.2277	0.059*	
C12	0.21445 (16)	0.22851 (18)	0.9941 (3)	0.0422 (5)	
H12A	0.1437	0.2176	0.9776	0.051*	
H3A	0.403 (2)	0.224 (2)	0.994 (3)	0.070 (9)*	

### Atomic displacement parameters (Å^2^)

**Table:**

	*U*^11^	*U*^22^	*U*^33^	*U*^12^	*U*^13^	*U*^23^
N1	0.0478 (11)	0.0662 (12)	0.0556 (12)	−0.0047 (9)	−0.0081 (9)	−0.0123 (11)
N2	0.0321 (8)	0.0543 (9)	0.0399 (9)	−0.0018 (7)	−0.0076 (7)	−0.0024 (8)
N3	0.0401 (8)	0.0488 (9)	0.0358 (9)	0.0008 (7)	−0.0086 (8)	−0.0013 (7)
O1W	0.0322 (7)	0.0646 (9)	0.0708 (11)	0.0027 (6)	0.0057 (8)	0.0108 (9)
O1	0.0562 (9)	0.0595 (9)	0.0491 (9)	−0.0023 (7)	0.0009 (8)	−0.0155 (8)
O2	0.0307 (7)	0.0630 (9)	0.0613 (10)	0.0010 (6)	−0.0087 (7)	−0.0070 (8)
O3	0.0354 (8)	0.1088 (15)	0.0502 (9)	0.0052 (8)	−0.0047 (7)	−0.0136 (11)
C1	0.0325 (9)	0.0358 (9)	0.0434 (10)	0.0001 (7)	−0.0015 (9)	0.0017 (8)
C2	0.0353 (10)	0.0559 (12)	0.0488 (12)	−0.0004 (8)	−0.0036 (9)	−0.0076 (10)
C3	0.0431 (11)	0.0424 (10)	0.0471 (12)	0.0001 (8)	0.0011 (10)	−0.0017 (9)
C4	0.0479 (13)	0.0867 (19)	0.080 (2)	0.0068 (12)	0.0167 (14)	−0.0125 (17)
C5	0.0350 (9)	0.0360 (9)	0.0420 (11)	0.0000 (7)	−0.0068 (8)	0.0030 (9)
C6	0.0377 (9)	0.0405 (9)	0.0388 (10)	0.0009 (7)	−0.0030 (8)	0.0058 (9)
C7	0.0386 (10)	0.0553 (11)	0.0390 (11)	0.0012 (8)	−0.0051 (9)	0.0017 (10)
C8	0.0486 (13)	0.0720 (16)	0.0528 (13)	−0.0036 (11)	−0.0138 (11)	−0.0078 (12)
C9	0.0750 (17)	0.0579 (14)	0.0420 (13)	0.0036 (11)	−0.0105 (12)	−0.0088 (11)
C10	0.0636 (15)	0.0623 (14)	0.0443 (12)	0.0138 (11)	0.0049 (12)	−0.0057 (11)
C11	0.0427 (10)	0.0545 (12)	0.0491 (12)	0.0040 (9)	0.0003 (11)	0.0015 (11)
C12	0.0355 (9)	0.0485 (11)	0.0426 (11)	−0.0002 (8)	−0.0068 (9)	0.0030 (9)

### Geometric parameters (Å, °)

**Table:**

N1—C2	1.294 (3)	C3—C4	1.476 (3)
N1—O1	1.405 (3)	C4—H4A	0.9600
N2—C5	1.341 (3)	C4—H4B	0.9600
N2—N3	1.372 (2)	C4—H4C	0.9600
N2—H2B	0.8600	C6—C11	1.393 (3)
N3—C12	1.274 (3)	C6—C7	1.406 (3)
O1W—H1W1	0.851 (17)	C6—C12	1.448 (3)
O1W—H1W2	0.831 (17)	C7—C8	1.386 (3)
O1—C3	1.338 (3)	C8—C9	1.377 (4)
O2—C5	1.224 (2)	C8—H8A	0.9300
O3—C7	1.353 (3)	C9—C10	1.368 (4)
O3—H3A	0.864 (18)	C9—H9A	0.9300
C1—C3	1.356 (3)	C10—C11	1.379 (4)
C1—C2	1.412 (3)	C10—H10A	0.9300
C1—C5	1.474 (3)	C11—H11A	0.9300
C2—H2A	0.9300	C12—H12A	0.9300
			
C2—N1—O1	105.15 (19)	O2—C5—C1	121.0 (2)
C5—N2—N3	118.78 (15)	N2—C5—C1	115.76 (15)
C5—N2—H2B	120.6	C11—C6—C7	118.2 (2)
N3—N2—H2B	120.6	C11—C6—C12	119.79 (19)
C12—N3—N2	118.15 (17)	C7—C6—C12	121.9 (2)
H1W1—O1W—H1W2	103 (2)	O3—C7—C8	118.0 (2)
C3—O1—N1	108.85 (18)	O3—C7—C6	122.3 (2)
C7—O3—H3A	109 (2)	C8—C7—C6	119.7 (2)
C3—C1—C2	103.6 (2)	C9—C8—C7	120.4 (2)
C3—C1—C5	126.24 (18)	C9—C8—H8A	119.8
C2—C1—C5	130.1 (2)	C7—C8—H8A	119.8
N1—C2—C1	112.6 (2)	C10—C9—C8	120.7 (2)
N1—C2—H2A	123.7	C10—C9—H9A	119.7
C1—C2—H2A	123.7	C8—C9—H9A	119.7
O1—C3—C1	109.82 (18)	C9—C10—C11	119.6 (2)
O1—C3—C4	116.5 (2)	C9—C10—H10A	120.2
C1—C3—C4	133.7 (2)	C11—C10—H10A	120.2
C3—C4—H4A	109.5	C10—C11—C6	121.4 (2)
C3—C4—H4B	109.5	C10—C11—H11A	119.3
H4A—C4—H4B	109.5	C6—C11—H11A	119.3
C3—C4—H4C	109.5	N3—C12—C6	120.83 (18)
H4A—C4—H4C	109.5	N3—C12—H12A	119.6
H4B—C4—H4C	109.5	C6—C12—H12A	119.6
O2—C5—N2	123.2 (2)		
			
C5—N2—N3—C12	−176.93 (18)	C2—C1—C5—N2	0.6 (3)
C2—N1—O1—C3	0.1 (2)	C11—C6—C7—O3	179.8 (2)
O1—N1—C2—C1	0.2 (2)	C12—C6—C7—O3	−2.7 (3)
C3—C1—C2—N1	−0.3 (2)	C11—C6—C7—C8	0.3 (3)
C5—C1—C2—N1	−178.83 (19)	C12—C6—C7—C8	177.8 (2)
N1—O1—C3—C1	−0.3 (2)	O3—C7—C8—C9	179.9 (2)
N1—O1—C3—C4	179.0 (2)	C6—C7—C8—C9	−0.6 (4)
C2—C1—C3—O1	0.3 (2)	C7—C8—C9—C10	0.9 (4)
C5—C1—C3—O1	178.94 (17)	C8—C9—C10—C11	−0.8 (4)
C2—C1—C3—C4	−178.7 (3)	C9—C10—C11—C6	0.6 (4)
C5—C1—C3—C4	−0.1 (4)	C7—C6—C11—C10	−0.3 (3)
N3—N2—C5—O2	−3.5 (3)	C12—C6—C11—C10	−177.9 (2)
N3—N2—C5—C1	175.77 (16)	N2—N3—C12—C6	−178.73 (16)
C3—C1—C5—O2	1.7 (3)	C11—C6—C12—N3	174.73 (19)
C2—C1—C5—O2	179.9 (2)	C7—C6—C12—N3	−2.7 (3)
C3—C1—C5—N2	−177.57 (19)		

### Hydrogen-bond geometry (Å, °)

**Table:**

*D*—H···*A*	*D*—H	H···*A*	*D*···*A*	*D*—H···*A*
O3—H3A···N3	0.87 (3)	1.89 (2)	2.617 (2)	145 (2)
N2—H2B···O1W	0.86	2.04	2.8847 (17)	169
O1W—H1W1···N1^i^	0.85 (3)	2.10 (2)	2.9304 (19)	166 (2)
O1W—H1W2···O2^ii^	0.83 (2)	1.96 (2)	2.7850 (17)	176 (2)

Symmetry codes: (i) −*x*, −*y*, *z*+1/2; (ii) *x*−1/2, −*y*+1/2, *z*.
